# Magnetoencephalography study of the Xingnao Kaiqiao acupuncture during acupuncture and language task states in healthy volunteers: a single-blind, randomized, sham-controlled trial protocol

**DOI:** 10.3389/fneur.2026.1876482

**Published:** 2026-08-18

**Authors:** Chun Sun, Feng Tao, Yiping Jiang, Mengxuan Lin, Siyi Jiang, Zhexu Dong, Saiwei Zhu, Haotian Wu, Yanan Zhang, Qiujian Meng, Yuying Jiang, Zhihong Meng, Yanlong Xie, Shizhe Deng, Jiangwei Shi

**Affiliations:** 1 First Teaching Hospital of Tianjin University of Traditional Chinese Medicine, Tianjin, China; 2 National Clinical Research Center for Chinese Medicine, Tianjin, China; 3 Beijing Quanmag Healthcare, Beijing, China; 4 Tianjin Academy of Traditional Chinese Medicine Affiliated Hospital, Tianjin, China; 5 Tianjin Academy of Traditional Chinese Medicine, Tianjin, China

**Keywords:** acupuncture state, healthy volunteers, language task, magnetoencephalography, Xingnao Kaiqiao acupuncture

## Abstract

**Background:**

The Xingnao Kaiqiao acupuncture protocol is a well-established technique in traditional Chinese medicine, widely used for neurological rehabilitation, particularly in patients with post-stroke aphasia. However, the central neural mechanisms underlying both single-acupoint and multi-acupoint needling within this protocol remain largely unclear. This study aims to use magnetoencephalography (MEG) to compare the cerebral magnetic signals evoked by verum and sham acupuncture of the Xingnao Kaiqiao protocol in healthy adults, and to explore the central mechanisms associated with language task states.

**Methods:**

This is a single-blind, randomized, sham-controlled trial. A total of 40 healthy, right-handed volunteers aged 18–45 years will be recruited and randomly allocated in a 1:1 ratio to either the verum acupuncture (VA) group or the sham acupuncture (SA) group. The VA group will receive needling at the standard Xingnao Kaiqiao acupoints, following the protocol’s established manipulation techniques. The SA group will receive superficial needling at non-acupoints located 1 cun lateral to each corresponding verum acupoint, without any manipulation. MEG data will be acquired during resting state, acupuncture state (single-acupoint and multi-acupoint sequences), and language task state (picture naming and language repetition tasks). Structural MRI data will be collected for source localization. The primary outcome will be changes in MEG signals before and after acupuncture. The secondary outcome will be the Chinese version of the Massachusetts General Hospital Acupuncture Sensation Scale (C-MASS).

**Discussion:**

This study will use MEG to explore the central effects of Xingnao Kaiqiao acupuncture at both single-acupoint and multi-acupoint levels. It may add to current understanding of how acupuncture affects brain networks related to language processing. The findings may provide a neuroimaging basis for further research on the mechanisms of this classical acupuncture technique.

**Clinical trial registration:**

International Traditional Medicine Clinical Trial Registry (https://itmctr.ccebtcm.org.cn/) ITMCTR2026000214. This study was registered on 14th January 2026.

## Background

Post-stroke aphasia (PSA) affects approximately 21 to 40% of stroke survivors and is among the most disabling long-term consequences of stroke for communication. It can substantially reduce quality of life and adversely affect rehabilitation outcomes (1). Speech and language therapy (SLT) remains the cornerstone of treatment for PSA. Better recovery has been reported with SLT delivered at higher frequency, particularly when therapy is function-oriented and includes both receptive and expressive language training. In most studies, effective treatment intensity is around 2 to 4 h per week, with a cumulative dose of more than 20 to 50 h (2). Intensive aphasia therapy has been shown to produce clinically meaningful gains in both acute and chronic stages. For example, the Queen Square Intensive Comprehensive Aphasia Program reported a large effect size for speaking improvement, and this benefit was maintained at 12-week follow-up (3, 4). Nevertheless, many patients continue to experience substantial language impairment despite intensive conventional rehabilitation. This persistent unmet need has prompted continued interest in adjunctive treatment strategies (5).

Among the adjunctive approaches being explored for PSA, acupuncture has received increasing attention (6). Evidence from systematic reviews and meta-analyses suggests that acupuncture, when combined with language rehabilitation training, may improve both overall language performance and specific language domains (7). Sang et al. reported significant improvements in auditory comprehension, spontaneous speech, repetition, and naming, with more pronounced effects during the subacute stage (8). Among the acupuncture approaches used in stroke rehabilitation, Xingnao Kaiqiao (XNKQ) acupuncture has been studied most extensively. Developed by Academician Shi Xuemin, it is a standardized protocol designed specifically for stroke rehabilitation and has been incorporated into International Technical Operation Standards (9). In our prior work, we demonstrated its clinical relevance through a multicenter, sham-controlled randomized clinical trial published in JAMA Network Open in 2024. In that study, XNKQ acupuncture produced a between-group difference of 7.99% on the Western Aphasia Battery Aphasia Quotient (WAB-AQ) at 6 weeks. This exceeded the minimal clinically important difference of 5.05%. The benefit remained evident at 6 months, with a between-group difference of 10.34% (10).

Despite these encouraging clinical findings, the neural mechanisms through which XNKQ acupuncture may support language recovery remain incompletely understood. Available neuroimaging studies suggest that acupuncture may influence brain oscillatory activity. Reported changes include reduced delta power and increased alpha power in ipsilesional frontal regions, and these alterations appear to be associated with functional recovery (11). Functional MRI studies have shown that acupuncture may affect activity and connectivity in classical language-related regions, including Broca’s and Wernicke’s areas (12). Acupuncture has also been associated with corticospinal tract remodeling, reflected by increased fractional anisotropy (13), and with broader changes in functional brain networks (14, 15), including the default mode network (16), sensorimotor network (17, 18), and cognitive control networks (19). However, most mechanistic work to date has relied on fMRI, which reflects hemodynamic responses rather than neuronal activity itself and therefore has limited temporal resolution. Much of the existing literature has focused on motor recovery, whereas language-related mechanisms remain less well characterized (20).

Magnetoencephalography (MEG) offers several advantages for addressing this gap. It records neuronal activity directly, provides millisecond temporal resolution, and achieves spatial precision of approximately 3 to 5 mm (21). It is sensitive to oscillatory activity across the delta, theta, alpha, beta, and gamma frequency bands. These characteristics make MEG well suited to examining how acupuncture may modulate dynamic neural activity within language networks (22, 23). Previous MEG studies have shown that acupuncture can induce frequency-specific changes in functional connectivity, including point-specific effects on temporal cortex connectivity. Manual acupuncture has been shown to reduce beta-band power in the contralateral primary somatosensory cortex (24, 25). Even so, most of these studies have been conducted under resting-state conditions or simple sensory paradigms, and language task-related brain activity during acupuncture has received much less attention (26).

Recent advances in optically pumped magnetometer-based MEG (OPM-MEG) have opened up new possibilities for this field. Compared with conventional SQUID-based MEG systems, OPM-MEG operates at room temperature, can be adapted more easily to different head sizes, and is more tolerant of participant movement during scanning. It may provide improved sensitivity and spatial resolution (27, 28). The availability of domestically developed OPM-MEG systems has improved the feasibility of this line of work (29). Current evidence suggests that OPM-MEG performs comparably to, and in some settings better than, SQUID-MEG across a range of neuroimaging paradigms. In particular, triaxial OPM configurations can capture approximately twice the total signal and provide better interference rejection than conventional single-axis systems (30–32).

Against this background, the present study will use OPM-MEG to investigate both resting-state and language task-related brain activity in healthy volunteers receiving XNKQ acupuncture. This work may help address an important gap in current mechanistic research. By comparing single-point and multi-point stimulation, the study will examine the neural basis of acupoint combination, as previous findings suggest that multi-point prescriptions may induce broader neural activation and engage additional neurochemical pathways than single-point stimulation (33, 34). The relationship between *deqi* sensation and MEG signals will also be examined. Earlier fMRI studies support this line of inquiry by showing that *deqi* is associated with activation in the limbic-paralimbic-neocortical network, including the thalamus, parahippocampal gyrus, insula, and cingulate cortex (35, 36). As a first step, this study characterizes the normal spatiotemporal patterns of cerebral magnetic responses to Xingnao Kaiqiao acupuncture and language tasks in healthy volunteers. Because these responses reflect the language-related networks targeted by acupuncture, they provide a normative reference for subsequent investigations in patients with post-stroke aphasia.

## Methods/design

### Study design and setting

**Figure 1:**
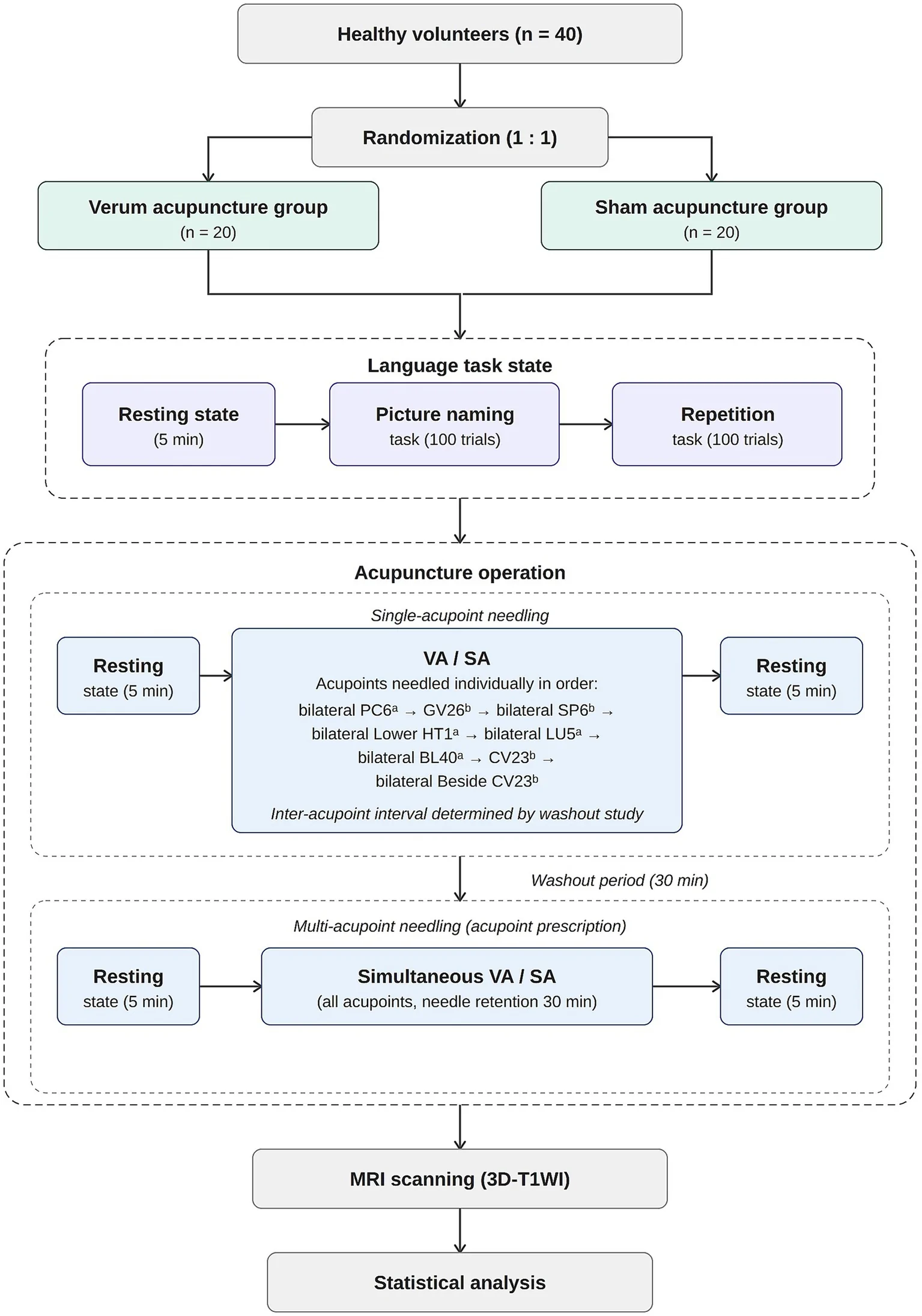
Flowchart of the trial. VA, verum acupuncture; SA, sham acupuncture; MRI, magnetic resonance imaging. **(a)** Immediate needling without retention. **(b)** Needle retention for 30 min with MEG recording during retention.

**Figure 2:**
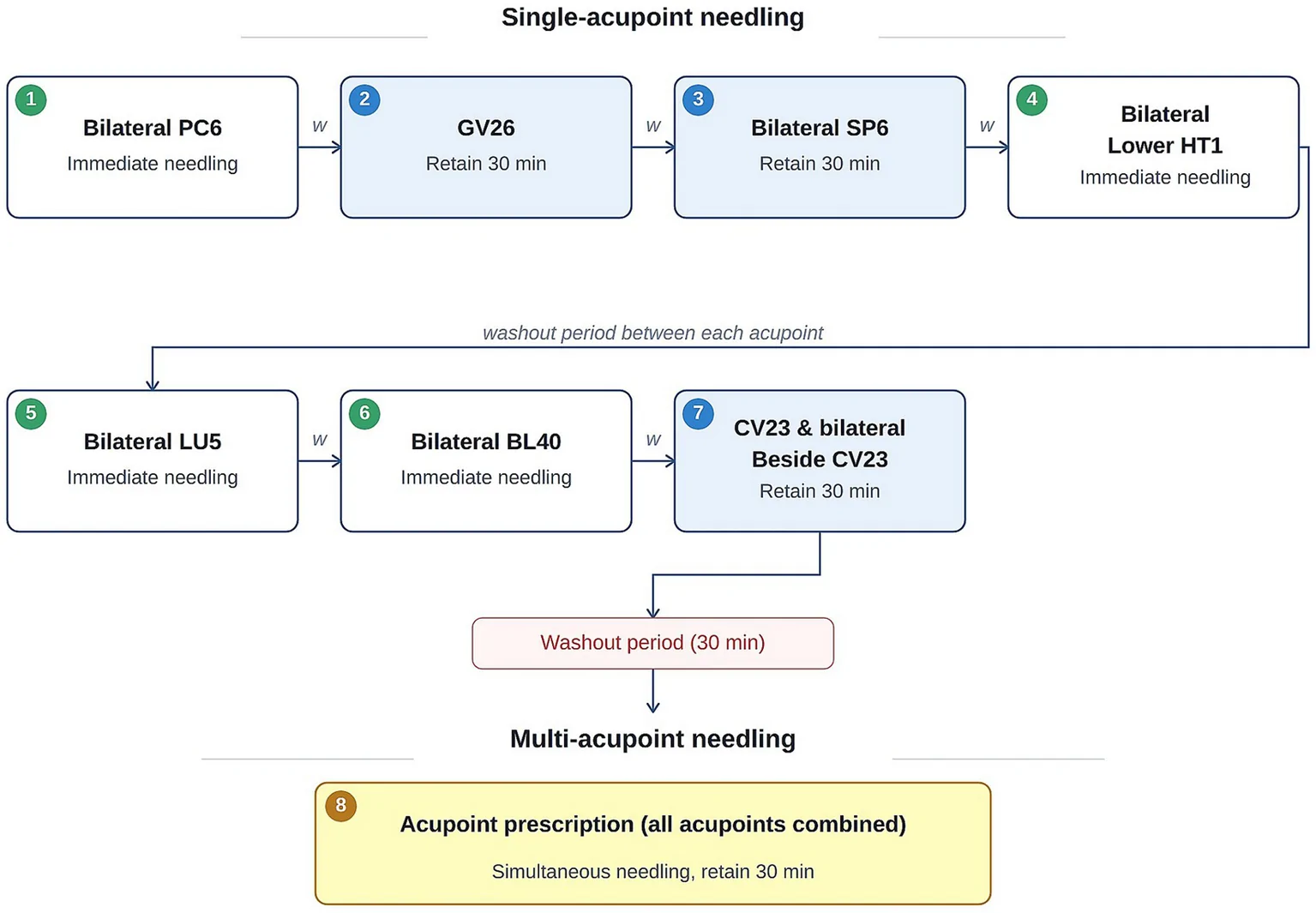
Acupuncture sequence. 1, 4, 5, 6: Immediate needling (no retention). 2, 3, 7: needle retention for 30 min. W: washout period.

This is a single-blind, randomized, sham-controlled trial designed to investigate the central neural mechanisms of the Xingnao Kaiqiao acupuncture using MEG. The study protocol adheres to the Standard Protocol Items: Recommendations for Interventional Trials (SPIRIT) guidelines. The study flowchart is presented in Figures 1, 2.

## Participants

### Recruitment

Eligible healthy volunteers will be recruited from the First Teaching Hospital of Tianjin University of Traditional Chinese Medicine. The study will be conducted from February 2026 to July 2026.

### Inclusion criteria

Participants who meet all of the following criteria will be eligible for inclusion:

1. aged 18–45 years;
2. right-handed;
3. no metallic or magnetic implants, and able to cooperate with MEG and MRI scanning;
4. no history of neurological, psychiatric, or severe medical conditions;
5. no claustrophobia;
6. no history of chronic pain or prior acupuncture treatment;
7. native Chinese speakers;
8. no acute upper respiratory infection, fever, severe rhinitis, cough, or other conditions that may affect scanning cooperation within 1 week prior to the experiment.

These criteria ensure a homogeneous sample of healthy adults without confounding neurological or medical conditions, while also ensuring safety and feasibility of MEG/MRI data acquisition (37, 38).

### Exclusion criteria

Participants meeting any of the following criteria will be excluded:

1. pregnant women;
2. individuals with extreme fear of acupuncture treatment;
3. recent use of central nervous system medications;
4. local skin infection at acupuncture sites;
5. tattooing, eyebrow tattooing, hair dyeing, or hair perming within the past month;
6. participation in other clinical trials within the past 3 months.

These exclusion criteria are designed to minimize confounding factors that could affect brain activity measurements and to ensure participant safety during acupuncture and neuroimaging procedures (39).

### Withdrawal criteria

Participants may withdraw from the study under the following circumstances:

### Participant-initiated withdrawal

1. Voluntary withdrawal at any time without providing a reason and without penalty.
2. Withdrawal due to intolerable discomfort or adverse events related to acupuncture or MEG/MRI procedures.
3. Non-compliance with study procedures (completion rate <80%).

### Investigator-initiated withdrawal

1. Development of serious adverse events that, in the investigator’s judgment, make continued participation inadvisable.
2. Discovery of previously unrecognized exclusion criteria (e.g., pregnancy, neurological condition).
3. Protocol violations that significantly affect data quality or participant safety.
4. Inability to complete MEG/MRI scans due to technical issues or participant factors (e.g., excessive head movement, claustrophobia).

### Sample size calculation

Currently, there is no unified standard for sample size calculation in MEG acupuncture research (37, 40). We will therefore enroll 40 healthy volunteers (20 per group). This sample size was informed by prior neuroimaging studies (41), in which 12–24 participants per group were used to examine brain activation differences between verum and sham acupuncture. Accounting for an anticipated 20% dropout rate, complete data are expected from at least 32 participants (16 per group).

### Randomization

Participants will be randomly assigned to the verum acupuncture (VA) group or the sham acupuncture (SA) group in a 1:1 ratio. The random allocation sequence will be generated using block randomization, stratified by sex, to ensure a balanced distribution of male and female participants across both groups. Allocation concealment will be maintained by having the research coordinator obtain the participant’s intervention assignment from sequentially numbered, opaque, sealed envelopes immediately before the first intervention session, and communicate this information exclusively to the acupuncturist.

### Blinding

A single-blind design will be employed, whereby participants will be blinded to their group allocation. In addition to participant blinding, the outcome assessors, MEG technicians, and data analysts will all be blinded to group assignment. Only the acupuncturist performing the needling will be aware of the allocation. At the end of the study, blinding success will be assessed by asking each participant to guess their group allocation (verum, sham, or “do not know”). Responses will be quantified using Bang’s Blinding Index (BI).

### Interventions

#### VA group

The VA group will receive acupuncture at the standard acupoints of the Xingnao Kaiqiao protocol. The acupoints include Neiguan (PC6, bilateral), Shuigou (GV26), Sanyinjiao (SP6, bilateral), XiaJiquan (Lower HT1, bilateral), Chize (LU5, bilateral), Weizhong (BL40, bilateral), Lianquan (CV23), and PangLianquan (Beside CV23, bilateral). Acupoint localization will follow the WHO Standard Acupuncture Point Locations in the Western Pacific Region (2020 edition). Disposable silver acupuncture needles (0.30 × 40 mm; Hwato Brand, Suzhou Medical Appliance Factory Co., Ltd., China) will be used. All acupuncture procedures will be performed by a licensed acupuncturist with at least 5 years of clinical experience.

**Figure 3:**
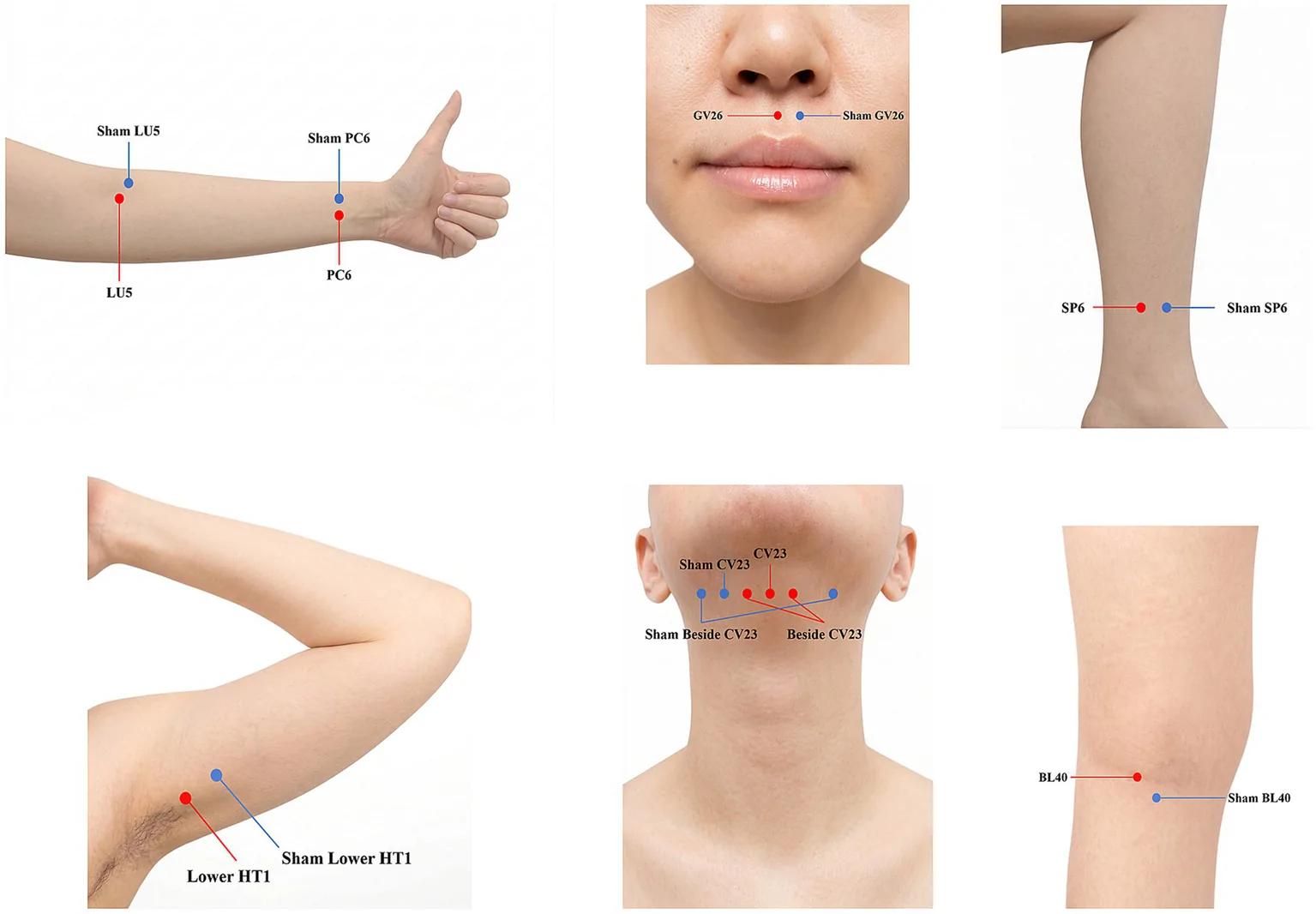
Location of verum acupoints and sham acupoints.

The acupuncture procedure will follow the standardized Xingnao Kaiqiao acupuncture protocol (42), with the CV23 and Beside CV23 techniques based on the research group’s prior study published in a JAMA Network Open randomized clinical trial (10). Participants will be positioned supine. The single-acupoint sequence will be performed first, followed by the multi-acupoint sequence. The acupuncturist will needle the points in the following order: Neiguan (PC6), Shuigou (GV26), Sanyinjiao (SP6), XiaJiquan (Lower HT1), Chize (LU5), Weizhong (BL40), Lianquan (CV23), and Beside CV23. The locations and needling techniques for each acupoint are described in Table 1 and Figure 3.

**Table 1:** Acupoint locations, needling techniques, and needle retention times in the VA group.

Acupoint name	Location	Needling technique	Retention
Neiguan (PC6, bilateral)	On the anterior forearm, 2 cun proximal to the palmar wrist crease, between the palmaris longus and flexor carpi radialis tendons	Perpendicular insertion 0.5–1.0 cun; twirling-rotating combined with lifting-thrusting reducing method; bilateral simultaneous manipulation for 1 min	No retention
Shuigou (GV26)	On the face, at the junction of the upper one-third and middle one-third of the philtrum	Oblique insertion 0.3–0.5 cun toward the nasal septum; sparrow-pecking reducing method until the eyes become moist or tearing occurs	30 min
Sanyinjiao (SP6, bilateral)	On the medial lower leg, 3 cun above the medial malleolus, posterior to the medial tibial border	Perpendicular insertion 1.0–1.5 cun; twirling-rotating reinforcing method; bilateral simultaneous manipulation for 1 min	30 min
XiaJiquan (Lower HT1, bilateral)	On the anterior upper arm, 1–2 cun inferior to Jiquan (HT1), along the line connecting Jiquan (HT1) and Shaohai (HE3)	Perpendicular insertion 1.0–1.5 cun; lifting-thrusting reducing method until 3 involuntary limb twitches are elicited	No retention
Chize (LU5, bilateral)	On the cubital crease, radial to the biceps brachii tendon	Perpendicular insertion 0.5–0.8 cun with elbow flexed at 120°; lifting-thrusting reducing method until 3 involuntary forearm twitches	No retention
Weizhong (BL40, bilateral)	At the midpoint of the popliteal crease, between the biceps femoris and semitendinosus tendons	Perpendicular or oblique insertion 1.0–1.5 cun with leg elevated and extended; lifting-thrusting reducing method until 3 involuntary limb twitches	No retention
Lianquan (CV23)	On the anterior midline, in the depression above the hyoid bone, superior to the thyroid cartilage	Perpendicular insertion 2.0 cun; lifting-thrusting and twirling-rotating to achieve *deqi*	30 min
PangLianquan (Beside CV23, bilateral)	0.5 cun lateral to Lianquan, in the depression above the notch of the thyroid cartilage	Perpendicular insertion 1.0–1.5 cun; lifting-thrusting and twirling-rotating to achieve *deqi*	30 min

#### SA group

Considering the practical constraints of the Xingnao Kaiqiao multi-acupoint protocol, in which spring-type placebo needles or skin-adhesive sham devices are not feasible and may compromise blinding, the SA control was designed based on published international acupuncture clinical trial protocols (10). The SA group will receive superficial needling at non-acupoints located 1 cun (approximately 20 mm, using the participant’s proportional measurement) lateral to each corresponding verum acupoint. The same type of needle (0.30 × 40 mm; Hwato Brand) will be used, and the procedure will be performed by the same qualified acupuncturist.

At each sham acupoint, the needle will be inserted superficially to a depth of 0.2–0.3 cun (approximately 4–6 mm), sufficient only to suspend the needle in the skin. No manual manipulation will be applied, and participants should not experience *deqi* sensation. Bilateral simultaneous needling and needle retention durations will be identical to the VA group. The single-acupoint sequence will be performed first, followed by the multi-acupoint sequence, consistent with the VA protocol.

### Assessment procedures

#### Washout period determination

Based on the research group’s pilot work, a washout period study will be conducted to determine the duration required for MEG signals to return to baseline following acupuncture. Shuigou (GV26) was chosen as the reference acupoint because it receives the strongest stimulation in the XNKQ protocol (sparrow-pecking reducing method until tearing) and is expected to produce the longest-lasting central responses. Using this point therefore yields a conservative estimate that should be sufficient for the other acupoints. MEG signals will be assessed at 0.5 h, 1 h, and 2 h post-acupuncture to evaluate the decay time of the acupuncture effect. The identified washout period will be used as the inter-acupoint interval during the single-acupoint sequence.

#### Assessment timepoints

The assessment will proceed in the following sequence. First, during the baseline period, participants will complete language tasks with simultaneous MEG recording. Second, a resting-state MEG scan will be performed after the language tasks and before the first acupuncture intervention. Third, the acupuncture state assessments will follow a sequence of single-acupoint needling first and then multi-acupoint needling.

In the single-acupoint sequence, after completing the language task and resting-state MEG scans, each acupoint will be needled individually, completing a total of seven acupuncture-state data acquisitions (CV23 and its auxiliary acupoints Beside CV23 are needled simultaneously and recorded as a single acquisition). For acupoints requiring 30-min needle retention (GV26, SP6, CV23, and Beside CV23), MEG data will be collected during the retention period, followed by a resting-state acquisition after needle removal. For acupoints without needle retention (PC6, HT1, LU5, and BL40), an immediate post-needling MEG acquisition will be performed. The interval between successive acupoints will follow the washout period determined in the pilot study.

In the multi-acupoint sequence, after all single-acupoint sessions are completed and the washout period has elapsed, a resting-state MEG scan will be performed. Subsequently, all acupoints will be needled simultaneously as a group, with MEG data collected during the retention period. After needle removal, a final resting-state MEG scan will be conducted to assess the overall central mechanism of the full Xingnao Kaiqiao acupuncture.

### MEG data acquisition

#### Language tasks

Prior to data recording, participants will be instructed to close their eyes, relax while remaining awake, focus their attention, and minimize head movement and facial muscle activity. A 5 min resting-state MEG recording will be collected. Each participant will then complete two cognitive tasks.

*Picture naming task*: Participants will view black-and-white line drawings from the Snodgrass and Vanderwart (S&V) database presented sequentially on a projection screen. Prior to the formal experiment, a pilot test was conducted among native Chinese speakers to assess picture-naming agreement, and only culturally familiar and unambiguous drawings with high naming agreement were selected for the experiment. They will be asked to name each picture verbally as quickly as possible following its appearance. The task consists of 100 trials with a randomized stimulus order, divided into two blocks of 50 trials with a short rest between blocks to reduce participant fatigue. Each trial begins with a central fixation cross presented for 500 ms, followed by the target picture presented for 1,500 ms. Participants are required to name the picture during or immediately after its presentation. The inter-trial interval is 3,000 ms. MEG signals will be recorded simultaneously throughout the task.

*Language repetition task*: Participants will listen to spoken words through earphones, selected from the Western Aphasia Battery (WAB) and the Chinese Rehabilitation Research Center Aphasia Examination (CRRCAE) repetition items. Prior to the formal experiment, a pilot test was conducted among native Chinese speakers to assess word familiarity, and only highly familiar and unambiguous items were selected for the experiment. All stimuli are spoken Mandarin Chinese with item length fixed at two characters and an acoustic duration of approximately 660 ms. They will be asked to repeat the auditory stimuli as quickly and accurately as possible after presentation. Each trial begins with a 500-ms fixation cue, followed by the target auditory stimulus, and then a 1,500–2,000 ms response window. The inter-trial interval is 1,000 ms. The task consists of 100 trials with simultaneous MEG recording, divided into two blocks of 50 trials with a short rest between blocks to reduce participant fatigue.

#### Resting-state recording

Each resting-state recording will last 5 min. To evaluate the effects of acupuncture intervention, each participant will complete one resting-state recording before and one after the acupuncture intervention.

#### Acupuncture-state recording

During the acupuncture task, participants will lie supine on the scanning bed, keeping their head stable and remaining as relaxed as possible. Before data collection, the investigator will explain the entire procedure and inform participants about the acupoint locations and needling sequence. All acupuncture procedures will be performed by a qualified acupuncturist according to the pre-determined protocol.

#### MEG acquisition parameters

**Figure 4:**
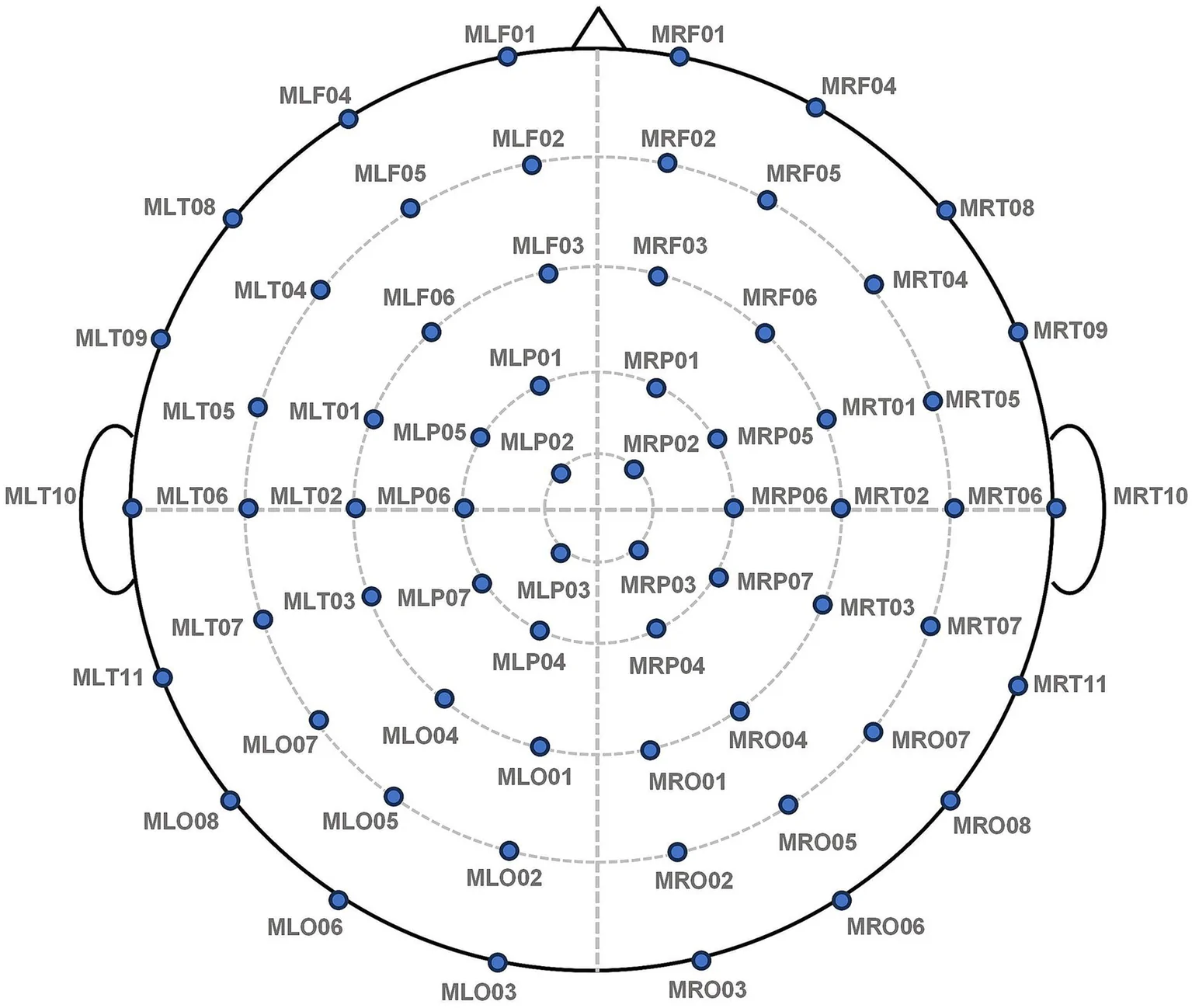
Spatial distribution of sensors within the MEG helmet.

MEG data will be acquired using the PyraMag Epoch 64 system (Quanmag Healthcare Co. Ltd., Beijing, China), equipped with 64 optically pumped magnetometer (OPM) sensors presented in Figure 4. The sampling rate will be set at 1000 Hz. The sensors are uniformly distributed across a helmet to achieve whole-brain coverage. Participants will lie comfortably on the scanning bed in a quiet environment. Prior to the formal acquisition, a 3D facial point cloud will be obtained for each participant using an optical scanning system (FreeScan Combo, Shining 3D Technology Co., Ltd., Hangzhou, China) for subsequent co-registration of OPM-MEG data with individual T1-weighted structural MRI images. After acquiring the facial point cloud, participants will be moved into the magnetically shielded cylinder to begin MEG recording.

#### MRI acquisition parameters

Structural MRI data will be acquired on a Siemens Skyra 3.0 T scanner using a 32-channel phased-array head coil. 3D T1-weighted images will be obtained using a magnetization-prepared rapid gradient echo (MPRAGE) sequence with the following parameters: repetition time (TR)/echo time (TE) = 1,900/2.2 ms; flip angle = 9°; slice thickness = 1 mm; matrix size = 256 × 256; field of view (FOV) = 256 × 256 mm^2^. During scanning, participants will be asked to close their eyes, relax, and remain awake. Earplugs will be provided to reduce noise, and foam padding will be used to minimize head motion.

### Outcome measures

#### Primary outcome

The primary outcome is the change in MEG signals before and after acupuncture intervention. MEG data will be collected during the resting state, acupuncture state, and language task state to evaluate the central effects of acupuncture. Specific MEG metrics include power spectral density (PSD) across different frequency bands (delta: 1–4 Hz, theta: 4–8 Hz, alpha: 8–13 Hz, beta: 13–30 Hz, gamma: 30–80 Hz), source-space neural activity, and whole-brain functional connectivity.

#### Secondary outcome

The secondary outcome is the C-MASS, assessed in both the VA and SA groups, which will be used to quantify participants’ subjective acupuncture sensations (*deqi*). The C-MASS scores will be used for subsequent correlation analysis with MEG metrics to explore the relationship between acupuncture sensation and central neural responses.

### Data analysis

#### MEG data preprocessing

MEG data will be preprocessed using the Brainstorm toolbox (based on MATLAB). First, zero-phase finite impulse response (FIR) notch filters will be applied at 50–250 Hz (in steps of 50 Hz) to remove power-line noise and its harmonics. A band-pass filter of 1–80 Hz will then be applied to retain frequencies of interest. The raw waveforms and power spectral density (PSD) plots of the preprocessed data will be visually inspected, and channels with poor data quality will be rejected. Bad channels will be interpolated using the mean signal from neighboring sensors within a 5-cm radius. Independent component analysis (ICA) will be used to remove physiological artifacts such as cardiac signals, and segments contaminated by muscle artifacts will be excluded from further analysis. Resting-state data will be segmented into 2-s epochs, and task-state data will be segmented into epochs from −500 ms to 1,500 ms relative to stimulus onset (time 0). Epochs containing artifacts will be manually rejected.

#### Source reconstruction

Individual anatomical MRI data will be processed using FreeSurfer for cortical surface reconstruction and brain tissue segmentation. Co-registration between MEG data and individual MRI will be performed using the facial point cloud. A forward model will be constructed using a boundary element model (BEM) based on each participant’s anatomical MRI. Source reconstruction will be performed using the dynamic Statistical Parametric Mapping method to obtain distributed source activity across the cortical surface.

#### Task-state analysis

For task-state data, brain activity will be extracted from event-related epochs spanning −500 ms to 1,500 ms relative to stimulus onset, with baseline correction applied from −200 ms to 0 ms. Source localization methods will be used to reconstruct brain activation at different time windows, thereby obtaining dynamic source-space responses during task execution. The analysis will focus on changes in activated brain regions during stimulus processing, phonological and language processing, and other task-related cognitive stages across different time points, thereby revealing the influence of acupuncture intervention on neural activity.

#### Resting-state analysis

For resting-state data, PSD values will be calculated for the delta (1–4 Hz), theta (4–8 Hz), alpha (8–13 Hz), beta (13–30 Hz), and gamma (30–80 Hz) bands. These values will be used to assess changes in spontaneous neural oscillatory activity after acupuncture. Source-level PSD will be analyzed to show the spatial distribution of spontaneous neural oscillatory activity. Functional connectivity in source space will be estimated with the phase locking index (PLI) to characterize large-scale brain network organization.

### Statistical analysis

For the resting-state PSD values, source-space neural activity, and whole-brain functional connectivity indices, both within-group and between-group comparisons will be performed. For within-group comparisons of pre- and post-acupuncture paired data, paired *t*-tests will be used. For between-group comparisons of the VA and SA groups, independent-sample *t*-tests will be employed. In whole-brain source-space statistical analyses, permutation-based cluster correction (≥5,000 permutations) will be applied to control for multiple comparisons and to identify brain regions with statistically significant differences.

For task-state data, time-window-based statistical methods will be used to compare brain activation intensity at different post-stimulus stages (time windows from 0 to 1,000 ms, in 100-ms steps). Linear mixed models will be applied to analyze condition effects and their interactions.

### Safety monitoring and adverse events

Safety evaluation will include general physical examination and assessment of adverse reactions. Potential adverse events related to acupuncture may include subcutaneous hematoma, minor post-needling bleeding, and needle fainting. Should any adverse events occur due to the procedure, appropriate examination and symptomatic treatment will be provided, and the participant and their family members will be informed accordingly.

All adverse events will be documented in detail, regardless of their perceived relationship to the study, including the time of onset, signs and symptoms, laboratory findings, management and outcomes, follow-up duration, severity, and duration. Concomitant medication use will also be recorded to facilitate analysis of the relationship between adverse events and the study intervention. Serious adverse events will be reported in compliance with institutional regulations.

### Data management and quality control

All raw MEG data, preprocessed data, and associated behavioral information will be stored following a standardized data management protocol. Data files will be anonymized using participant identification numbers, and all personally identifiable information will be removed to ensure data security and privacy protection. All data will be stored on encrypted servers with multi-level access controls and regular backups to prevent data loss. Preprocessing pipelines, analysis scripts, and parameter settings will be recorded using version control systems to ensure reproducibility and traceability.

### Ethics and dissemination

This study has been approved by the Ethics Committee of the First Teaching Hospital of Tianjin University of Traditional Chinese Medicine. Written informed consent will be obtained from all participants prior to enrollment. Participants will be free to withdraw from the study at any time without penalty. Study results will be disseminated through peer-reviewed publications and presentations at academic conferences.

## Discussion

### Treatment advances and research bottlenecks in acupuncture for post-stroke aphasia

Despite accumulating evidence supporting acupuncture for post-stroke aphasia, major research bottlenecks remain. In our previous work, a sham-controlled trial (10) published in 2024 demonstrated clinically meaningful improvements with XNKQ acupuncture that were sustained through 6 months. However, the broader literature (43, 44) is still frequently constrained by small sample sizes and methodological limitations, such as inadequate blinding and heterogeneous outcome measures. In addition, optimal acupuncture parameters have yet to be defined, including point selection, stimulation intensity, treatment frequency, and treatment duration. Crucially, the central neural mechanisms through which acupuncture modulates language function remain incompletely understood, thereby limiting rational protocol optimization and the development of patient selection strategies (12).

### Rationale for studying healthy volunteers

This mechanistic study was conducted in healthy volunteers to reduce sources of variation that are difficult to control in stroke populations. After stroke, neural responses are shaped by lesion location, lesion size, and time since onset, and patterns of language network reorganization can differ substantially across individuals (23, 45). This makes it difficult to separate acupuncture-related effects from lesion-related changes and spontaneous recovery. In healthy participants, brain responses to acupuncture can be examined under more stable conditions and with fewer confounding influences, such as stroke-related brain injury, medication use, and disease-related neural reorganization. This may allow clearer identification of neural signatures associated with acupuncture and may also help define dose–response relationships between stimulation parameters and brain activity (46). In addition, studies in healthy volunteers allow tighter experimental control, repeated measurements, and more systematic adjustment of acupuncture parameters than is usually feasible in patient populations (47). Findings from healthy participants may therefore provide a useful reference for later studies in patients.

### Advantages and challenges of OPM-MEG in acupuncture research

To our knowledge, this is the first study to apply OPM-MEG to the investigation of acupuncture mechanisms. OPM-MEG provides millisecond temporal resolution, which makes it possible to track the time course of brain responses during acupuncture. Neural effects can therefore be examined across different timescales, from immediate responses during needle manipulation to more sustained changes after needle removal (25, 26). Unlike hemodynamic imaging methods such as fMRI (48), MEG records neuronal currents directly rather than secondary vascular responses. This offers a more direct view of how acupuncture influences neural activity. It is particularly relevant in acupuncture research, where the timing of neural events may be important for understanding mechanism and where hemodynamic responses may not fully reflect the underlying neural process. OPM-MEG also has practical advantages over conventional SQUID-based systems. The closer sensor-scalp distance may improve sensitivity and spatial resolution. The system can also be adapted more easily to different head sizes and is more tolerant of participant movement during scanning (28, 29). These features are useful for acupuncture studies, where participant comfort and relatively natural experimental conditions are important for eliciting authentic neural responses and *deqi* sensations.

Several challenges should also be considered. MEG is mainly sensitive to tangential cortical sources and is less effective for detecting activity in deep brain structures such as the thalamus and basal ganglia, which are relevant to language processing and may also be influenced by acupuncture (21). In previous MEG studies of acupuncture, variation in study design, analytical methods, and sample size has contributed to inconsistent findings (47). Standardized MEG acquisition and analysis protocols for acupuncture research are not yet established. In addition, the relationship between MEG findings and clinical outcomes still requires further validation (49). Multimodal approaches combining MEG and fMRI may therefore provide a more complete account by drawing on the strengths of both methods (50).

### Language task paradigms in acupuncture mechanism research

The use of language tasks together with MEG provides a useful approach for studying how acupuncture affects language-related neural networks. Tasks such as picture naming and word repetition engage core components of language processing, including lexical-semantic access, phonological encoding, and speech motor programming. They also produce reliable activation in known language regions, including Broca’s area, Wernicke’s area, and the pathways connecting them (51). Compared with resting-state approaches, task-based neuroimaging has several advantages. It probes neural systems directly relevant to aphasia, helps clarify how acupuncture influences neural processing during active language production and comprehension, and allows comparison of acupuncture-related effects across different language domains (22, 52). Recent work suggests that language recovery is multidimensional. Fluency, semantic-executive function, and phonology may follow different recovery trajectories and rely on partly distinct neural substrates. Acupuncture-related effects may therefore differ across language components. This possibility can be examined by combining multiple language tasks with MEG recording (53, 54).

### Single-point versus multi-point effects in XNKQ acupuncture

An important question in this study is whether acupoint combinations produce effects beyond those of individual points. Neuroimaging work by Zhang et al. showed that stimulation at LR3 combined with KI3 activated a broader set of brain regions than stimulation at LR3 alone. Additional activations were found in the posterior cerebellar lobe and Brodmann area 6 (BA6), and BA6 was uniquely engaged under the combined LR3 plus KI3 condition (33). In animal studies, Jiang et al. reported that electroacupuncture at ST36 and GB30 produced stronger analgesic effects through activation of the endocannabinoid system, whereas this effect was not observed with single-point stimulation (34). Building on these findings, the present study compares neural activation during single-point stimulation with that observed during the complete XNKQ formula. This comparison may help identify formula-specific activation patterns, clarify the relative contribution of individual points to language network modulation, and explore possible synergistic effects within acupoint combinations. It may also inform future refinement and personalization of the XNKQ protocol.

### *Deqi* sensation and MEG signal correlation

In traditional Chinese medicine theory, therapeutic effects are closely linked to *deqi*, a complex sensation that may include soreness, numbness, distension, and heaviness (55). Neuroimaging studies have shown that *deqi* is associated with activity in brain regions involved in sensory, affective, and contextual processing. These include the thalamus, postcentral gyrus, insula, cingulate cortex, and parahippocampal gyrus, with effects extending to the limbic-paralimbic-neocortical network and the default mode network (56). Graded fMRI studies have also shown that stronger acupuncture stimulation can produce larger responses in sensorimotor processing regions, with both psychophysical and psychophysiological responses increasing as stimulation intensity increases (46). Most previous work, however, has relied on fMRI and cannot capture the rapid temporal dynamics of *deqi*-related neural activity. In the present study, MEG will be used to record immediate neural responses associated with *deqi* onset. The study will also examine whether *deqi* is linked to changes in specific neural oscillations and whether activity in particular brain regions correlates with *deqi* intensity and quality, as measured by the C-MASS (57). Examining *deqi* together with MEG signals during language tasks may also help clarify whether *deqi* is related to modulation of language-relevant brain activity.

### Strengths and limitations

This study has several strengths. To our knowledge, it is the first to apply OPM-MEG to the study of acupuncture mechanisms. It uses a multi-state design that includes resting-state, language task-state, and acupuncture-state recordings. It also compares single-point and multi-point stimulation in a systematic way. The study builds on a rigorous clinical trial that has already shown therapeutic effects of XNKQ acupuncture. In addition, it uses validated language tasks that are directly relevant to aphasia and includes key methodological safeguards such as randomization, blinding, and sham acupuncture controls.

Several limitations should also be acknowledged. First, the study is conducted in healthy volunteers rather than stroke patients, which limits direct clinical translation. Second, it focuses on immediate neural responses and cannot address longer-term effects over days or weeks, which may be more relevant to treatment outcomes. Third, the sample size of 40 participants is acceptable for an MEG study but remains small compared with large clinical trials. Fourth, the study is specific to the XNKQ protocol and may not generalize to other acupuncture approaches. Fifth, the limited sensitivity of MEG to deep brain structures may restrict characterization of the full distributed effects of acupuncture. Finally, only a limited set of language tasks is included, and therefore effects on other language functions such as sentence comprehension and discourse production were not be assessed. Individual differences in brain anatomy, acupuncture sensitivity, and *deqi* experience may also introduce heterogeneity at the group level.

### Implications for future clinical research

The findings of this study may help inform later clinical research on XNKQ acupuncture for post-stroke aphasia. Brain regions and networks identified as responsive to acupuncture in healthy volunteers may provide a basis for more targeted investigation in patient populations. In particular, these normal patterns can serve as a baseline for interpreting acupuncture-related responses in patients. Deviations from this baseline may then help define hypotheses and analysis parameters for subsequent patient studies. Differences between single-point stimulation and the complete XNKQ formula may also help guide future refinement of the protocol. If *deqi* is found to be associated with specific neural responses, this may support more precise adjustment of acupuncture parameters in clinical studies. The MEG patterns identified here may also have potential value in future work on patient stratification or outcome prediction, although this will require further validation. More broadly, the multi-state MEG framework used in this study may be useful for subsequent mechanistic research on acupuncture and other neuromodulatory interventions. A better understanding of how acupuncture influences language-related networks may also help optimize the timing of combined acupuncture and SLT.
